# Acceptability, cost-effectiveness, and capacity of a facility-based seasonal influenza vaccination among high-risk groups: a study protocol in selected tertiary care hospitals of Bangladesh

**DOI:** 10.1186/s12889-024-17724-6

**Published:** 2024-01-20

**Authors:** Md Zakiul Hassan, Md Abdullah Al Jubayer Biswas, Mahbubur Rahman, Homayra Rahman Shoshi, Ashrak Shad Pyash, Md Ariful Islam, Md Azizul Haque, Syeda Rukhshana Parvin, Md Tanvir Hossen, Mofakhar Hussain, Mahmudur Rahman, Tahmina Shirin, Fahmida Chowdhury

**Affiliations:** 1 Programme for Emerging Infections, Infectious Disease Division, International Centre for Diarrhoeal Disease Research, Bangladesh (icddr,b), Mohakhali, Dhaka, Bangladesh; 2 Nuffield Department of Clinical Medicine, University of Oxford, Oxford, UK; 3 Institute of Epidemiology, Disease Control and Research, Mohakhali, Dhaka, Bangladesh; 4 The Expanded Programme on Immunization (EPI), Maternal Neonatal Child and Adolescent Health of the Ministry of Health & Family Welfare of Bangladesh, Dhaka, Bangladesh; 5 Department of Medicine, Rajshahi Medical College, Rajshahi, Bangladesh; 6 Department of Paediatrics, Khulna Medical College, Khulna, Bangladesh; 7 Institute of Health Policy, Management and Evaluation, University of Toronto, Toronto, Canada; 8 Global Health Development (GHD), The Eastern Mediterranean Public Health Network (EMPHNET), Abdallah Ben Abbas St, Building No. 42, Amman, Jordan

**Keywords:** Seasonal influenza vaccination, Vaccination cost-effectiveness, High-risk group population, Vaccine acceptability, Health belief model

## Abstract

**Background:**

In Bangladesh, seasonal influenza imposes considerable disease and economic burden, especially for those at high-risk of severe disease. The most successful approach for influenza prevention is the administration of a vaccine. Many poor and middle-income nations, including Bangladesh, do not have a national strategy or program in place for seasonal influenza vaccines, despite the World Health Organization’s (WHO) advice to prioritize high-risk populations. Additionally, there is a scarcity of substantial data on the cost-effectiveness of seasonal influenza vaccination in these countries. The aim of our study is to determine acceptability, health beliefs, barriers, and intention of receiving influenza vaccine among high-risk populations, assess the cost-effectiveness of implementing a facility-based seasonal influenza vaccination programme, and investigate the required capacity for a potential seasonal influenza vaccination programme.

**Methods:**

We will undertake this study following STROBE guidelines. We will conduct the study in inpatient and outpatient departments of three selected tertiary-level hospitals leveraging the ongoing hospital-based influenza surveillance (HBIS) platform. The study population will include the WHO-defined four high-risk groups excluding healthcare workers: children six months to eight years, pregnant women, elderly ≥ 60 years, and adults with chronic diseases. We will collect quantitative data on participants’ acceptability, health beliefs, barriers, and vaccination intentions using the health belief model (HBM) from patients meeting the criteria for high-risk populations attending two public tertiary-level hospitals. In one of the two public tertiary-level hospitals, we will arrange an influenza vaccination campaign before the influenza season, where the vaccine will be offered free of cost to high-risk patients, and in the second hospital, vaccination will not be offered. Both the vaccinated and unvaccinated participants will then be followed-up once a month for one year to record any influenza-like illness, hospitalization, and death. Additional data for objective two will be collected from patients with symptoms of influenza-like illness (ILI) and severe acute respiratory infection (SARI) at one public and one private hospital to determine both direct and indirect costs associated with influenza illness. We will estimate the required number of influenza vaccines, safe injections, and total storage volume utilizing secondary data. We will use a deterministic Markov decision-analytic model to estimate the cost-effectiveness of facility-based influenza vaccination in Bangladesh.

**Discussion:**

The results of this study will enable the National Immunization Technical Advisory Group and the Ministry of Health & Family Welfare of Bangladesh to decide what steps to take to develop and implement an influenza vaccination strategy targeting high-risk populations.

**Trial registration:**

The Clinicaltrials.gov registration number is NCT05996549. The registration for the protocol version 2.0 took place in August 2023, with the initial participant being enrolled in March 2022.

## Background

Globally, Seasonal influenza is a major contributor to morbidity and mortality, resulting in significant health and economic consequences [1–3]. Based on data provided by the World Health Organization (WHO), it is estimated that influenza affects approximately one billion individuals annually [4]. Among these cases, 3 to 5 million individuals experience severe illness, resulting in 290,000 to 650,000 annual fatalities [4]. Low- and middle-income countries (LMICs) bear a disproportionately higher burden of influenza-related illness in comparison to high-income countries (HICs) [5]. Within LMICs, certain demographic groups are particularly vulnerable to experiencing severe illness as a result of influenza [5]. These groups include pregnant women, children under the age of five, the elderly, healthcare workers, and adults who have underlying health conditions [5]. Previous reports showed that older people were at higher risk of death and hospital admission during flu season [6]. The report also found that pregnant women and children were at higher risk of developing pneumonia and requiring hospital admission; the adults with co-morbidity conditions were at risk of hospitalization and intensive care admission during flu seasons [6].

To minimize the risk of influenza infection and reduce the severity of the associated illness, the World Health Organization (WHO) recommended in 2012 that persons at high risk of experiencing severe influenza symptoms receive an annual dosage of the influenza vaccine [7]. Despite the WHO recommendation of seasonal influenza vaccination prioritizing high-risk groups, 76% of the LMICs, including Bangladesh, lack a national policy/programme on seasonal influenza targeting high-risk groups [8, 9]. Notably, the WHO African Region (6.3%) and the WHO South-East Asian Region (18%) had the lowest proportions of countries having influenza vaccine policies [9].

Moreover, influenza vaccine coverage remains low in LMICs. A systematic review of seasonal influenza vaccine coverage in LMICs revealed considerable variation in vaccination rates across different demographic groups [10]. Specifically, among children, the coverage ranged from as low as 2% to as high as 72% in these countries [10]. Similarly, the vaccination coverage among the elderly ranged from 10 to 70%, while pregnant women exhibited a range of 0–4% coverage [10]. Among healthcare professionals, the vaccination coverage varied from 20 to 56% in the LMICs under study [10]. The potential cause for the low vaccination coverage may be attributed to the suboptimal rates of influenza vaccine uptake [11–14]. Research conducted on populations at high risk for influenza revealed varying rates of influenza vaccine uptake [11–14]. Specifically, the uptake of the influenza vaccine was observed to be 33.3% among healthcare workers, 5.4% among elderly individuals, 27% among pregnant women, and 33.7% among adults with co-morbid conditions [11–14].

To identify challenges to promoting influenza vaccinations in LMICs, several studies explored various barriers to influenza vaccination, such as inadequate infrastructures to provide universal vaccination services and insufficient systems for evaluating, procuring, regulating, storing, distributing, monitoring, and administering the vaccine [15, 16]. Also, the lack of context-specific knowledge on the economic burden of influenza vaccines on high-risk groups, lack of medical socio-behavioral patterns, and socio-economic consequences of influenza have been identified as challenges for promoting influenza vaccinations in LMICs [15, 16].

In Bangladesh, the influenza-associated mortality rate was estimated at six per 100,000 for under-five children and 41 per 1000,00 for persons > 60 years [17]. Also, the rate of hospitalization associated with influenza was estimated to be between 4.4 and 6.7 per 1,000 individuals in the population [18]. In contrast, the rate of influenza among outpatients with influenza-like illness (ILI) was estimated to be between 100 and 170 per 1,000 individuals in the population [18]. In 2010, approximately 25 million individuals across all age groups sought outpatient medical care for influenza in Bangladesh [3]. This resulted in an annual direct expenditure of US$ 108 million specifically attributed to influenza-related outpatient visits [3]. Moreover, approximately 30,592 laboratory-confirmed influenza patients of all ages were hospitalized each year, with an estimated annual influenza-associated hospitalization cost of US$ 1.4 million [3].

Influenza vaccines are the most reliable method for preventing influenza infection. Unfortunately, Bangladesh does not have a national influenza vaccination program among the WHO-recommended high-risk population [19]. The Ministry of Health and Family Welfare (MoH&FW), the Government of the People’s Republic of Bangladesh, provides complimentary influenza vaccinations exclusively to Hajj pilgrims [20]. This service is offered in adherence to the obligatory criteria set by the Saudi Government for all individuals participating in the Hajj pilgrimage [20]. However, there is a lack of information to drive the MoH&FW policy on influenza vaccinations in high-risk populations.

For reducing the influenza disease burden, influenza vaccination has demonstrated significant efficacy and cost-effectiveness, particularly within populations at high risk of infection [21, 22]. The immune responses induced by influenza vaccines are typically specific to the particular viral strain. The presence of antibodies specific to a certain type or subtype of influenza virus typically offers minimal or no immunity against other types or subtypes. Furthermore, such antibodies usually do not protect against antigenic variants of the same virus that emerge due to antigenic drift. Vaccination can potentially induce a “back boost” of antibody titers specifically targeting influenza A(H3N2) viruses in the adult population. Influenza vaccines are administered on an annual basis due to the occurrence of antigenic drift and shift. The efficacy of these vaccines, which typically ranges between 40% and 60%, exhibits considerable variation across different settings and influenza seasons [10]. In addition, the findings of a systematic review indicated that out of the 118 studies examined, 22 studies demonstrated that influenza vaccination resulted in cost savings [23]. This study’s findings also stated that there were 13 studies reporting cost-effectiveness ratios of $10,000 per outcome, another 13 reporting ratios ranging from $10,000 to $50,000 per outcome, and three reporting ratios equal to or exceeding $50,000 per outcome [23]. Although the cost-effectiveness of influenza vaccination has been established, it is necessary to note that the applicability of cost-effectiveness parameters may vary across different countries due to variations in influenza disease profiles, unit costs of vaccination, and health system delivery mechanisms [24]. Hence, it is imperative to have country-specific estimates to allocate limited resources effectively.

Bangladesh lacks context-specific data on the acceptability, cost-effectiveness, and vaccination capacity needed for influenza vaccination among the WHO-defined high-risk population, which hinders informed vaccination strategies.

### Objectives

We propose to generate preliminary data on high-risk groups’ health beliefs, barriers, and intent to receive the influenza vaccine. We will also generate preliminary data on the acceptability, cost-effectiveness, and required capacity for facility-based influenza vaccination for informing policy decisions regarding influenza vaccination among the high-risk population in Bangladesh.

## Methods

This study will employ a quasi-experimental design that aligns with the STROBE guidelines. Additionally, this design will incorporate sensitivity analyses to explore the impact of uncertainty in parameter estimation precision on the outcomes of deterministic Markov decision-analytic models.

### Study design

We will conduct our study leveraging the ongoing HBIS platform, a network of nine tertiary-level hospitals across Bangladesh [19, 25]. The structure and operational aspects of the HBIS platform have been previously discussed [19, 25]. Our study design will be quasi-experimental. To meet the vaccine acceptability objective, we will collect data on participants’ health beliefs, barriers, and vaccination intentions using the health belief model (HBM) from patients meeting criteria for high-risk populations attending two public tertiary-level hospitals. To meet the influenza vaccination cost-effectiveness objective, in one of the two hospitals, we will conduct an influenza vaccination campaign before the influenza season, where the vaccine will be offered free of cost to high-risk patients, and in the second hospital, vaccination will not be offered. Both the vaccinated and unvaccinated participants will then be followed-up once a month for one year to record any influenza-like illness, hospitalization, and death. Additional data for vaccination cost-effectiveness objectives on direct and indirect costs associated with influenza illness will be collected from ILI and SARI patients at one public and one private hospital. To meet the required capacity for a facility-based influenza vaccination objective, we will collect secondary data, including the Statistical Yearbook of Bangladesh 2020, as represented in Table 1 [26–28].

**Table 1:** Assumptions and data sources by each target group

Target Group	Assumption and data sources
Children 6 months to 8 years, influenza vaccine doses	Two doses will be delivered, each four weeks apart [31, 32]. Data of the total number of < 5 years children will be adopted from the Statistical Yearbook of Bangladesh 2020 [26]. Monthly the number of children < 8 years visited hospitals will be collected from respected hospitals or respective District Civil Surgeon Office [29, 30, 33].
Pregnant women’s influenza vaccine doses	One dose to all pregnant women (any trimesters). The crude birth rate is assumed to be 21.9% (BDHS 2017-18) [34]. Influenza vaccines were distributed evenly across the childbearing years 15 through 49 [34]. Data on the total number of women of childbearing age (15–49 years) will be collected from the Statistical Yearbook of Bangladesh 2020 [26]. Monthly pregnant women who visited hospitals will be collected from respective hospitals or respective District Civil Surgeon Office [29, 30, 33].
Elderly ≥ 60 years influenza vaccine doses	One dose for all persons aged 60 years and older. Population data from the Statistical Yearbook of Bangladesh 2020 [26]. Monthly the total number of ≥ 60 years aged patients visited hospitals will be collected from respected hospitals or respective District Civil Surgeon Office [29, 30, 33].
Adults with chronic diseases, influenza vaccine doses	One dose for all persons with chronic disease. We will use the prevalence of having any chronic illness from the Bangladesh Household Income and Expenditure Survey (HIES) 2010 [35]. Then, the total number of persons with any chronic illness will be estimated using data from the Statistical Yearbook of Bangladesh 2020 [26]. Monthly patients with chronic diseases visited hospitals will be collected from respected hospitals or respective District Civil Surgeon Office [29, 30, 33].

**Fig. 1:**
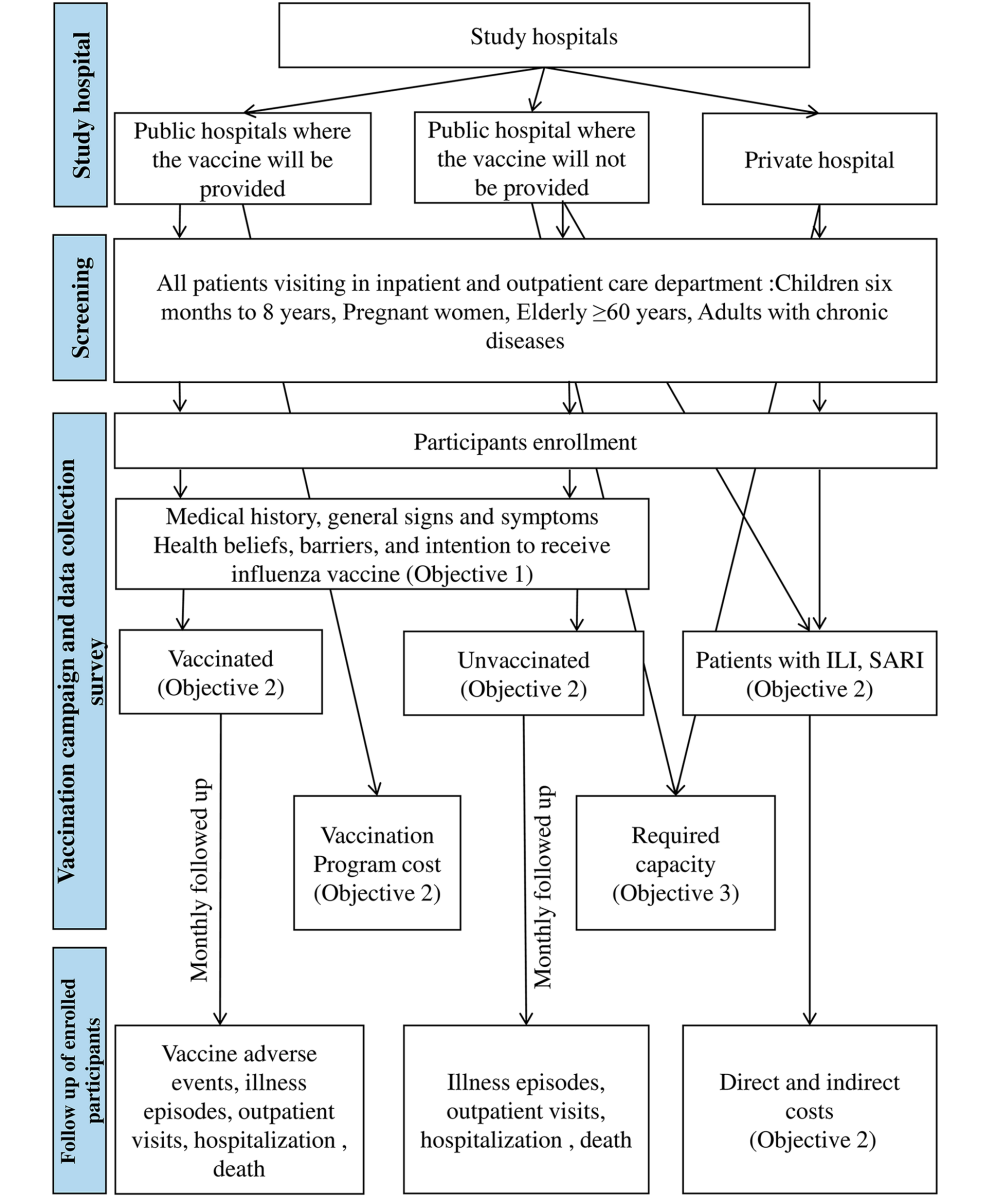
Flow chart showing different elements of study activity and data collection

We will conduct all the study activities through close collaboration with the Institute of Epidemiology Disease Control and Research (IEDCR) of the Ministry of Health and Family Welfare (MoH&FW), the Government of Bangladesh. We will also collaborate closely with the administration of the participating hospitals, especially the hospital director, to guarantee the hospitals’ participation, support, and cooperation. We will work with the line director and directors of study hospitals to implement the vaccination. We will utilize hospital resources and leverage existing staff to vaccinate study participants. Figure 1 illustrates the study activities and data collection flow chart.

### Study setting

We selected study hospitals considering several factors. Firstly, we will use the hospital-based influenza surveillance (HBIS) platform to conduct this study. We have chosen Rajshahi Medical College Hospital and Khulna Medical College Hospital for our study activities on high-risk groups’ vaccine acceptability and cost-effectiveness. We will enroll Severe acute respiratory illness (SARI) and influenza like illness (ILI) patients identified from the HBIS platform to meet our study objectives. We will also collect influenza virus test results from the HBIS platform and data on influenza illness episodes’ costs from the influenza-positive SARI and ILI patients. This approach will save influenza virus testing costs not allocated in the current study budget. Secondly, to assess the outcome of influenza vaccination, we plan to enroll patients from public tertiary-level hospitals (one treatment/vaccinated and the other control hospital) to minimize baseline differences between the treatment/vaccinated and control arm. Thirdly, we have chosen Ragib-Rabeya Medical College & Hospital to represent public and private healthcare facility cost differences while estimating the costs of influenza illness. A brief description of the study sites’ bed occupancy rate and average monthly admission are represented in Table 2 [29, 30].

**Table 2:** Demographic characteristics of the study hospitals

Study sites	Bed occupancy rate (%)	Total no OPD visits	Total number of PNC visits	Total number of admission	Middle wealth quantile
Rajshahi Medical College Hospital	198.05	114,818	2,405	22,022	24.1%
Khulna Medical College Hospital	275.00	35,311	991	8,859	24.8%
Jalalabad Ragib-Rabeya Medical College & Hospital	NA	NA	NA	NA	15.8%

Source: Local health bulletin October 2022

OPD = outpatients care department; PNC = post-natal visit

Note: a bed occupancy rate greater than 100% indicates that these hospitals receive and care for more patients than their capacity

Study sites for influenza vaccine acceptability, health beliefs, barriers:

We will conduct a health belief model (HBM) survey in two conveniently selected hospitals:

1. Rajshahi Medical College Hospital, Rajshahi.
2. Khulna Medical College Hospital, Khulna.

**Fig. 2:**
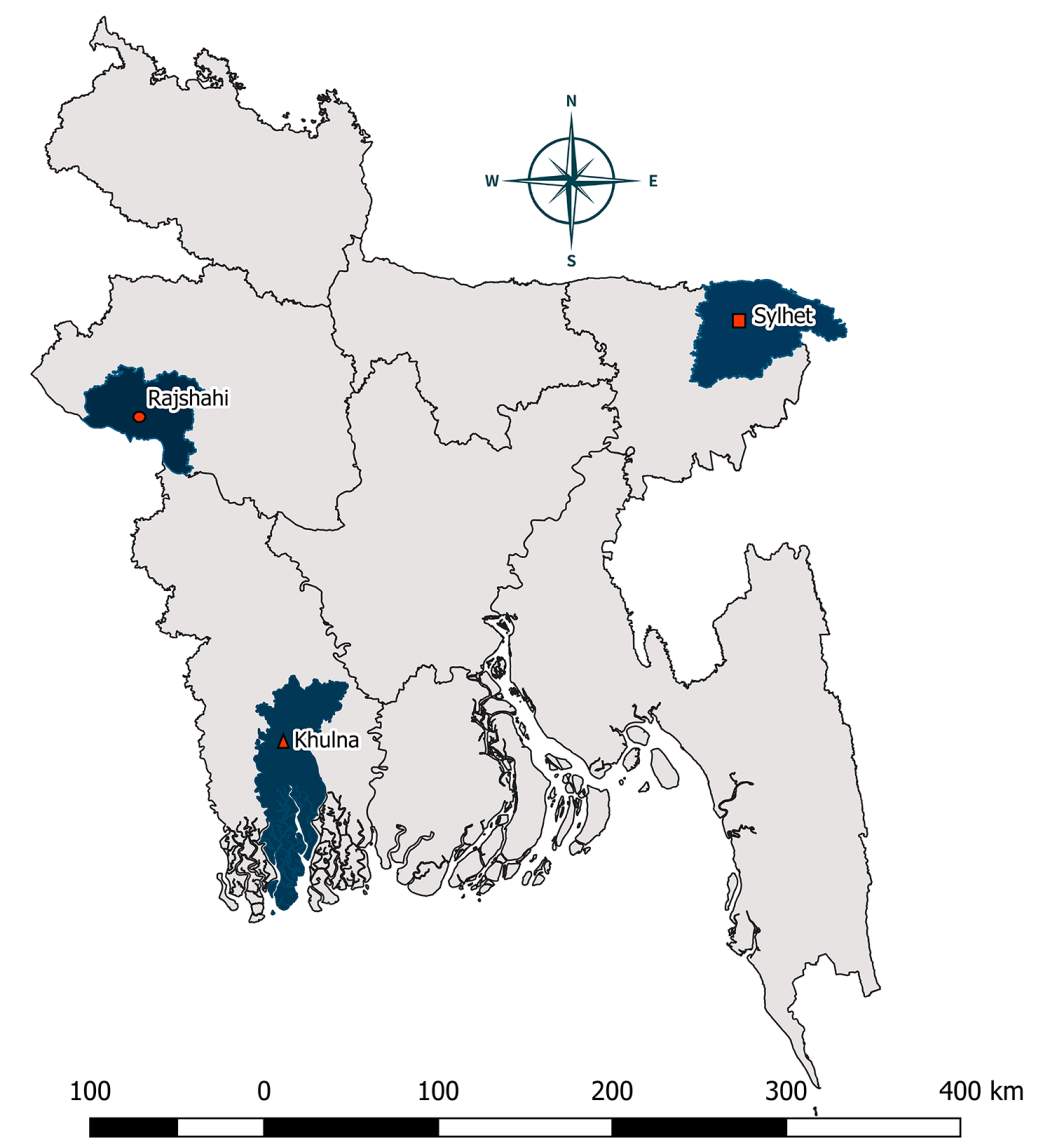
Location of the study hospitals; red ellipse represents Rajshahi Medical College Hospital, Rajshahi; red rectangle represents Jalalabad Ragib-Rabeya Medical College & Hospital; red triangle represents Khulna Medical College Hospital

Study sites for influenza vaccination cost-effectiveness objective:

For this specific objective, we will collect data on the health outcome of vaccination and the cost associated with influenza illness. Data on health outcomes will be collected from two public tertiary-level hospitals. The cost associated with influenza illness data will be collected from one public and one private hospital. Figure 2 represents the study sites.

Health outcomes of vaccination:

1. Rajshahi Medical College Hospital, Rajshahi.
2. Khulna Medical College Hospital, Khulna.

Cost associated with influenza illness:

1. Rajshahi Medical College Hospital, Rajshahi.
2. Jalalabad Ragib-Rabeya Medical College & Hospital, Sylhet.

Study sites for required capacity for a facility-based influenza vaccination objective:

We will utilize secondary data to estimate the required capacity for seasonal influenza vaccination at three hospital catchment areas, as illustrated in Table 1.

### Study population and participant recruitment

The study population will consist of persons belonging to the four high-risk groups as defined by the World Health Organization (WHO), except for healthcare workers: children six months to eight years, pregnant women, elderly ≥ 60 years, and adults with chronic diseases [7]. We have excluded healthcare workers from our targeted study population to prevent duplication with our prior research efforts [19]. We will enroll study participants who meet specific inclusion and exclusion criteria. Table 3 depicts the criteria utilized to determine the inclusion and exclusion of participants in our study.

**Table 3:** List inclusion and exclusion criteria

Inclusion criteria	Exclusion criteria
Participants will be visiting study hospitals (outpatients and inpatients only. Not emergency departments) for routine care during the study period.	Participants with severe, life-threatening allergies to any ingredient in a flu vaccine (other than egg proteins)
Participants who will be children six months to eight years, or aged more than 60 years, or pregnant women (any trimester), or have any chronic disease such as chronic respiratory disease (requiring regular medication), diabetes, chronic renal disease, any cancer, excluding basal and squamous skin cancers, immune suppression or immune deficiency, chronic liver disease, chronic hematological disorder, chronic kidney disease, chronic neurological impairment/disease, serious mental health condition, organ or bone marrow recipient, history of any prior surgery, percutaneous coronary intervention, high blood cholesterol, family history of cardiovascular disease, other pre-existing conditions.	Participants having a history of severe allergic reactions (e.g., anaphylaxis) after a previous dose of influenza vaccine or to a vaccine component.
Participants /parents/caregivers who will be willing to sign the informed consent form	Participants having a history of Guillain-Barre syndrome less than six weeks after a previous dose of influenza vaccine.
Participants/parents/caregivers who will have an operational mobile phone number and are responsible for sharing updated phone numbers in case of plans to change for the next year	Participants having a history of moderate or severe acute illness with or without fever. The vaccine should be given after the acute condition has improved.
Participants/parents/caregivers who will have fixed addresses with no plan to leave the address for the next one year	
Participants/parents/caregivers will be aware of the follow-up schedule and understand the study expectations during the follow-up.	

### Variables

We thoroughly reviewed the literature on different outcome variables and explanatory factors in our research [5, 31, 36–44]. Regarding our first objective, which centered on vaccine acceptability, our primary outcome of interest wil be the high-risk groups’ intention to receive the influenza vaccination. This will assist us in assessing the propensity of high-risk groups’ willingness to receive influenza vaccine. Our second objective focuses on vaccination effectiveness, precisely measuring vaccine efficacy against influenza in high-risk group groups. This metric will enable us to assess the efficacy of the vaccination in protecting high-risk group groups. In addition, we will thoroughly examine the cost components related to influenza, focusing on the overall costs of influenza-related illnesses in high-risk groups as our second objective’s variables. To understand the complex factors that affect vaccine acceptance and effectiveness, we will examine various explanatory variables, including age, gender, occupation, location, marital status, and income, among others.

### Data sources and measurement

#### Data collection to assess health beliefs, barriers, and intention to receive an influenza vaccine

We will collect data on acceptability, health beliefs, obstacles, and current intentions to receive the vaccine from enrolled participants (Fig. 2). We will capture these data using the health belief model (HBM). The questionnaire has been developed using an existing literature review [36–41, 45]. We will include items to assess five theoretical constructs of HBM based on reviewing relevant literature, including high-risk individual’s perceived susceptibility to disease, perceived severity of influenza, perceived benefits of the vaccine, perceived barriers to the vaccine, cues to action, and self-efficacy [37, 38, 46]. We will conduct face-to-face interviews for the data collection.

### Data collection to determine vaccination cost-effectiveness

#### Vaccination campaign

To record health outcomes after influenza vaccination, in one of the two public tertiary-level study hospitals, we will conduct an influenza vaccination campaign before the influenza season, where the vaccine will be offered free of cost to high-risk patients, and in the second hospital, vaccination will not be offered. We will work with the hospital administration to set up a vaccination booth on the hospital premises. Vaccines will be made available at the vaccination booths. Posters and leaflets containing information on influenza vaccination will be displayed at the vaccination booths and key hospital locations with information on the vaccination. Inpatients and outpatients of all departments will be informed about the ongoing immunization campaign. Eligible patients will be offered to take the vaccine during the vaccination campaign. Benefits, significance, adverse effects, and how to manage them will be explained to the patients. Nurses/ Health Assistants (HA) will provide influenza vaccination throughout the campaigns. At 8 AM, the vaccination booth will open and close at 2 PM, six days a week throughout the campaign period. A vaccination card will be issued to the participants. All high-risk groups will get one dose of seasonal influenza vaccination (Quadrivalent), except children aged six months to 8 years, who will receive two doses four weeks apart [31, 32]. All vaccines will be the southern hemisphere vaccine as recommended by the WHO for Bangladesh. Regarding children, we will make phone follow-ups with the guardians of the children receiving the first dose to remind them about the second dose schedule, and it will be recorded in the vaccine card if the second dose is received. In order to ensure the availability and quality control of vaccines throughout the three-month campaign period, the research team at icddr,b will closely monitor the campaign. All vaccinated participants will receive notification regarding potential adverse events following immunization (AEFI) during the vaccination process. To document any untoward occurrences after Adverse Events Following Immunization (AEFI), we will adhere to the pre-existing surveillance mechanism implemented by the World Health Organization (WHO) and the Ministry of Health and Family Welfare (MoHFW) of the government of Bangladesh. During the vaccination campaign briefing session in each study hospital, participants will be informed about the process of reporting any Adverse Events Following Immunization (AEFI) within the hospital’s established surveillance system. Additionally, participants will be provided with the mobile number, as stated in the consent form, to report any such issues. Furthermore, during the vaccination campaign implementation, a representative from icddr,b will be assigned to each study hospital to oversee and monitor any adverse events following immunization (AEFI) in conjunction with the hospital’s existing surveillance system. If an Adverse Event Following Immunization (AEFI) case is identified, the personnel affiliated with icddr,b will promptly notify the Hospital Surveillance Officer (HSO), provide support to the HSO in completing the AEFI report form, and subsequently transmit it to the Expanded Programme on Immunization (EPI) headquarters via the Chief Health Officer.

In the second hospital, we will enroll participants meeting the high-risk group individual criteria. However, no vaccination campaign will be conducted. We will name this group as an unvaccinated cohort.

### Follow-up of vaccinated and unvaccinated participants

icddr,b field staff will follow up with vaccinated and unvaccinated study participants bi-weekly through mobile phone for the entire influenza season. During the follow-up, data will be recorded on health outcomes, including respiratory symptoms, influenza-like illness, hospitalization, and death. To minimize loss to follow-up, icddr,b field team will contact the cohort member at their convenience. icddr,b field team will use the alternative number collected during enrolment where they cannot reach their regular mobile phone number. At the end of one follow-up interview, the interviewee will be consulted about the convenient time of their upcoming follow-up date. After taking these approaches, if it is impossible to reach a cohort member for three follow-up schedules, he/she will be declared lost to follow-up case. For the loss to follow-up case, icddr,b field team will place notes on their data identification number. The study flow chart is shown in Fig. 2.

### The cost associated with influenza illness

For collecting data on costs associated with influenza illness, we will enroll high-risk individuals visiting study hospitals who will meet the SARI and ILI case definitions:

Severe acute respiratory illness (SARI): In the case of patients across all ages, the presence of a history of or a recorded temperature equal to or exceeding 38.0 °C and cough began during the last ten days and required hospitalization [47].

Influenza-like illness (ILI): Measured fever above ≥ 38.0 °C with cough having an onset within the last ten days [47].

We will collect data on the enrolled participants’ direct and indirect medical costs associated with influenza illness episodes. Cost breakdowns for influenza illness episodes are provided below.

### Direct medical and non-medical costs for influenza illness episodes

Direct costs will consist of health care provider fees, hospital registration fees, bed rental, prescriptions, laboratory testing, transportation, and mobile phone calls. We will also record informal payments made during hospital visits. All medications, laboratory tests, registration, and room rental costs will also be included. We will also collect payment if patients visit a pharmacy or other clinics prior to hospitalization. The non-medical expenses for patients or caregivers include food, lodging, and transportation. Our field staff will collect data directly from patients and their families. Members of the participant’s family or the participants themselves will be able to determine the drugs and tests they received at no cost due to hospital subsidies and those for which they incurred personal expenses (out-of-pocket).

### Productivity loss or indirect cost

We will also collect data on participants’ or caregivers’, or family members’ lost productivity and indirect costs incurred during illness. We will record the number of workdays missed by participants’ family members, participants themselves, and caregivers due to sickness or family caregiving. We will exclude weekends and national holidays from our calculation. Every day missed due to illness or the need to care for sick family members will be considered a forfeited workday for hourly-wage workers and housewives. We will not consider the days missed due to decreased activity, such as a partial work day, absences from school, or fatalities caused by influenza. The number of productive days lost due to influenza-related fatalities will be estimated based on the baseline mortality rate. This will be multiplied by the median daily wage in Bangladesh, followed by an adjustment based on the prevailing unemployment rate in the country.

### Costs of durable equipment and other fixed costs

We will collect the primary data on the costs of the supplies and materials consumed (not purchased and stored) at the hospitals involved in diagnosing and treating influenza illness by department, ward, and service, for example, per unit cost and required units for blood drawn, x-ray, blood culture test, C-reactive protein, widal test, complete blood count, etc. We will also collect data related to health facility operating hours (working hours by ward and department), the ticket prices of different departments’ inpatient or outpatient units, and bed rent.

### Collating secondary data for estimating cost-effectiveness of vaccination

#### Disability-adjusted life year (DALY)

We will collect secondary data on disability-adjusted life years (DALY) from the WHO global health estimation [48]. We will utilize age, and symptoms-specific DALY estimates to determine the DALY associated with influenza-related health outcomes. Though QALY is widely used for hospital patients, due to the unavailability of publicly available QALY data related to influenza or influenza-like illnesses for LMICs, we have decided to use the WHO’s publicly available DALY estimates for our study. We will utilize the WHO-recommended information spreadsheet. The spreadsheet contains estimates for disability-adjusted life year (DALY), categorized by WHO region, cause, age, and sex. The term “regional classification” pertains to the World Health Organization’s regional groupings as of 2019, which aligns with the most up-to-date reference year for this Global Health Estimates revision. The compilation of these statistics was carried out by the Department of Data and Analytics at the World Health Organization (WHO) in partnership with various technical programs within the WHO. The cause-specific estimates may exhibit significant ranges of uncertainty depending on the data sources that are accessible [49, 50]. The WHO Department of Data and Analytics prepared these statistics in collaboration with WHO technical programs [51, 52]. The cause-specific estimates may exhibit significant ranges of uncertainty depending on the data sources that are accessible. Due to alterations in data and some methods, these estimates are not comparable to previously released WHO estimates. Similar literature has been identified about facility-based vaccination, wherein the DALY metric was employed to assess the cost-effectiveness of such programme [53, 54].

#### Data sources for estimating influenza-associated disease burden

To estimate the key parameters of influenza-associated disease burden, such as the number of outpatient visits, hospitalization, and deaths, we will collect data from secondary sources [17, 26, 31, 34, 35, 54–58]: Table 1 represents the assumption and secondary data sources by each target risk group.

#### Vaccine coverage

We will use secondary data for the input parameters relating to seasonal influenza vaccination coverage in Bangladesh [10, 59–61]. We will use our proposed study data on the number of targeted high-risk group individuals getting influenza vaccination during the immunization campaign to estimate vaccine coverage [62]. We will extract information on hospitals’ catchment areas’ population size from the Statistical Yearbook of Bangladesh 2020 [26].

#### Vaccine adverse event costs

Vaccine adverse event costs will be estimated for each targeted risk group. Using primary and secondary data, we will estimate the costs of influenza vaccination adverse events. We will use secondary data to estimate influenza vaccination rates and adverse events, whereas primary data will be used to determine the cost [34]. We will assume that patients who have a minor adverse event will seek medical care at an outpatient facility where they will receive consultation from a qualified physician. On the other hand, all patients who experience a severe adverse event will need hospitalization. The unit cost for minor adverse events will be determined by the price charged by the outpatient care department and the fee charged by the physician. Fees for outpatient care departments and healthcare professionals will be derived from primary data. Similarly, the estimation of the cost associated with a severe adverse event involves the calculation of inpatient care costs, which encompass both general ward facility fees and specialist fees. This estimation is derived by multiplying these costs by the length of the patient’s hospital stay. The average duration of hospital stay for influenza-associated illnesses will be collected from secondary data, and inpatient care daily costs will be collected from primary data [63].

#### Influenza vaccination programme cost

The financial and economic costs associated with the vaccination programme will be assessed through the utilization of the World Health Organization’s Seasonal Influenza Immunization Costing Tool (SIICT) [64]. The WHO SIICT was updated in 2020 and was newly named “The WHO Flutool Plus” [64]. The newly developed WHO Flutool Plus enables the estimation of costs associated with various vaccination activities, including microplanning, procurement, distribution, training, social mobilization, service delivery, supervision, and the acquisition of supplemental cold chain equipment [64]. We will customize the Flutool Plus as required to estimate the cost of our facility-based vaccination programme. During the vaccination campaign, icddr,b research personnel will keep track of all cost components of the influenza vaccine campaign. The cost components of the vaccination campaign at the facility level that will be recorded are shown in Table 4.

**Table 4:** Cost components of the facility-based piloted influenza vaccination campaign

Dimension	Components of costs
**Start-up cost**	
Micro-planning	• Personnel time spent in meetings • Per diems and travel allowances • Venue rental • Transport
Training	• Value of personnel time spent on training • Development of training materials • Per diems and travel allowances • Venue rental • Transport • Training materials • Stationery
Social mobilization/IEC	• Value of personnel and volunteer time spent on material development and other activities • Facilitator time in meetings • Per diems and travel allowances • Stationery • Printing of posters and leaflets • Production of TV and/or radio spots
**Recurrent or operational costs**	
Procurement of vaccines and injection supplies	• Cost of vaccines and injection supplies, regardless of the source of financing • Cost of freight, clearance, insurance, and taxes
Service delivery	• Value of personnel time spent on vaccination • Transport fuel • Personnel per diems to travel to vaccination sites • Supplies– e.g. cotton
Monitoring and evaluation	• Tally sheets or registers • Pens and pencils • Vaccination cards • Materials for vaccine booths
Supervision	• Value of personnel time spent on supervision • Travel allowances • Transport fuel and maintenance • Stationery
Waste management	• Purchase of incinerators (annualized/discounted) • Fuel • Transport
**Capital costs**	
Cold chain equipment	Cold chain equipment (annualized and discounted)

### Data sources for determining the required capacity of facility-based influenza vaccination

We will estimate the required capacity of the seasonal influenza vaccination programme based on the cold chain capacity requirements of the hospitals. We will also collect secondary data to calculate requirements at each hospital.

Secondary data will be collected about the population size of the targeted risk group in the hospital’s catchment area, the number of doses required for vaccinating each high-risk individual, the number of doses per vial, packed volume, doses syringes, and safety box [26–30]. Table 1 represents the assumptions on the population size of the targeted risk group in the catchment area.

## Study size

### Sample size and sampling

#### The sample size for the vaccine acceptability objective

Our study design will be quasi-experimental. Our primary outcome for vaccine acceptability is the intention to receive the influenza vaccine. Based on studies utilizing the high-risk group-specific health belief model conducted in neighboring countries, it has been found that 91% of elderly people, 88% of adults with chronic illness, 76.3% of pregnant women, and 62.4% of parents with six to three-year-olds expressed their intention to get the influenza vaccine [38–40, 45]. We expect a similar positive intention to receive the influenza vaccine, ranging from 62 to 91% for our target study population. In this study, we employed a significance level of 5%, a power of 80%, a design effect of 1.2, and account for a non-response rate of 10% to determine the required sample size [65–67]. We will utilize a validated health belief model tool. To avoid selection bias, we will use random assignment to the study based on predefined inclusion and exclusion criteria. We will also provide training to study staff about random enrolment of the study participants based on inclusion and exclusion criteria. Table 5 represents the estimated sample size by the high-risk group for the vaccine acceptability objective.

**Table 5:** Estimated sample size for investigating the vaccine acceptability/ intention to receive, health outcome due to vaccination, and cost-effectiveness of implementing a facility-based influenza vaccination

High-risk group	The sample size for the vaccine acceptability survey	The sample size for vaccine effectiveness	The sample size for the cost-components survey
Children 6 months to 8 years	659	465	60
Pregnant women	495	756	39
Elderly ≥ 60 years	330	736	56
Adults with chronic diseases	425	610	31

#### The sample size for the vaccine effectiveness objective

Our primary outcome for health outcome for vaccination is vaccine efficacy against influenza among high-risk group populations. Studies from neighboring countries found that ILI symptoms were 15.30% of children, 31.23% of adults with chronic illness, 10.97% of pregnant women, and 20% of the elderly [68–70]. A systematic review of seasonal influenza vaccine policy, use, and effectiveness also revealed that pooled vaccine effectiveness for high-risk groups in the tropics and subtropics ranged from 48 to 88% (10). We expect a similar vaccine efficacy between 50 and 81% for our study’s targeted high-risk group population. A 5% significance level, 80% power, design effect 1.2, and 10% non-response were used to calculate the required sample size to estimate vaccine effectiveness [65–67]. Table 5 represents the high-risk group’s estimated sample size for vaccine effectiveness.

#### The sample size for the cost-components survey

Our primary outcome for costs-components is the total influenza-associated illness costs among high-risk group populations. A study conducted in Bangladesh examined the economic assessment of diseases associated with influenza [3]. The findings indicated that the overall direct costs related to influenza-induced illness were estimated at USD 70 (7,614 Taka) for children under the age of five [3]. Pregnant women incurred costs of USD 191 (20,808 Taka), while the elderly faced expenses of USD 126 (13,761 Taka) [3]. Adults with chronic diseases had direct costs amounting to USD 75 (8,222 Taka) [3]. Our study anticipates a comparable aggregate direct cost of influenza-related illness ranging from USD 70 (7,614 Taka) to USD 191 (20,808 Taka) for the specific population of high-risk individuals we targeted. Other than that, our study employed a significance level of 5%, a power of 80%, a design effect of 1.2, and accounts for a non-response rate of 10% to determine the appropriate sample size [65–67]. Table 5 represents the high-risk group’s estimated sample size for vaccine effectiveness. For both of our objectives, we will screen and enroll study participants from both inpatient and outpatient across all clinical departments of the study hospitals, who will be seeking routine care and treatment at the study hospitals.

#### Quantitative variables

We will conduct a thorough literature review to help us choose the quantitative variables for our study, drawing upon a range of sources [5, 31, 36–44]. The variables will be categorized into three broad categories. Firstly, we will encompass sociodemographic variables, which consist of a wide range of sociodemographic data related to the specific high-risk group being studied. Secondly, in the medical history section, we included variables regarding the medical history of individuals at high risk and any common signs and symptoms they experienced in the previous 30 days. The third category includes direct and indirect cost components related to using medical resources for SARI and ILI. These variables include expenses that occur before, during, and after receiving treatment for SARI and ILI. This provides a thorough understanding of the economic factors associated with these conditions.

## Statistical methods

We will summarize all the data using various descriptive statistics tools, such as frequency, percentage, mean, median, standard deviation (SD), and interquartile range (IQR, calculated as the difference between the 25th percentile and the 75th percentile).

### Objective specific data analysis plan is as follows

#### Data analysis on the propensity to vaccine using results from the vaccine acceptability survey

In each of the five HBM constructs, we will use factor analysis using the principal axis factor technique and varimax rotation to find latent variables and minimize the number of independent variables. The Kaiser-Meyer-Olkin test will be used to determine sample adequacy. The parameters that suggest an acceptable fit on the factor analysis will be the goodness of fit index (GFI) and root mean square error of approximation (RMSEA) [71]. We will also use Cronbach’s alpha to assess the internal consistency of survey items [45]. We will use the regression models with robust error estimation to predict and understand the association between influenza vaccination status and all five HBM constructs.

### Data analysis for cost-effectiveness analysis

#### Vaccine efficacy estimation

We will utilize Poisson regression to calculate the rate ratios (RRs) of participants who will receive influenza vaccination compared to those who will not. In the final model, we will include all the variables that will incorporate all variables that exhibit a confounding effect or demonstrate statistical significance at a significance level of 5%. The investigation of additional potential effect modifiers will be conducted by incorporating interaction terms. We will calculate vaccine effectiveness with 95% CI for each risk group ( (1 - RR) * 100) [72].

#### Cost-effectiveness estimation

To estimate the cost-effectiveness of our pilot influenza vaccination porgramme targeted for the high-risk group, we will use the built-in formulas of the CETSIV tool. The CETSIV is a Microsoft Excel-based tool designed to analyze the cost-effectiveness of influenza vaccination using a decision tree model [43]. It features built-in formulas for deterministic Markov decision-analytic models to evaluate the incremental costs and DALY of the vaccination compared to a controlled scenario [43]. Besides, we will recheck the outputs by developing a deterministic Markov decision-analytic model to simulate outcomes under alternative assumptions of input parameters. Individual DALYs will be calculated for each risk group’s individual using the WHO global health estimates for DALYs [49]. Incremental cost-effectiveness ratios (ICERs) will be calculated as the ratio of incremental cost over incremental DALYs. We will conduct a probabilistic sensitivity analysis (PSA) to see how parameter estimation precision uncertainty affects the model’s outcome. Since we will develop a cohort-based Markov model, each model had different years of estimation depending on the age at which each cohort enters the mode. Hence, such calculated ICERs will be measured using different future time frames. In order to ensure consistency of ICERs across all cohorts, the discount will be applied to ICERs using the number of years remaining life years. We will assume a life expectancy of 75 and a discount rate of 5%. Identified key parameters will change simultaneously over predetermined probability distribution. The Markov model will be run 1,000 times for each risk group, assuming variations in key parameters using pre-defined probabilities to allow for uncertainty in parameters. If ICER is below less than three times the gross domestic product (GDP) per capita, we will consider the vaccination as cost-effective, as per the WHO standard [73–76]. Also, we will estimate rates of influenza cases averted /outpatient visits averted/hospitalization averted/ deaths averted, or influenza treatment cost or productivity loss averted by influenza vaccination in each risk group using the method described by Meredith et al. 2019 [77].

### Data analysis to calculate the required capacity of facility-based influenza vaccination

We will estimate annual influenza vaccine dose and safe-injection equipment requirements for the target hospitals, adopting the WHO guidelines to calculate vaccine volumes and cold chain capacity [78, 79]. We will also estimate the required storage volume, shortage volume for the vaccine, and safe injection [78, 79]. We will compare the anticipated vaccine volumes to the vaccine cold storage capacity estimates to assess the hospital’s operational feasibility.

### Vaccination cost estimation

We will be using the Flutool Plus add-on for Excel. Flutool Plus calculates each cost type’s financial and economic costs and displays the results. Besides Flutool Plus calculation, we will also calculate each cost type’s financial and economic costs for cross-checking purposes. To do so, we will multiply the data input quantities by the financial and economic unit costs to get the incremental financial and economic costs. However, the Flutool Plus tool’s default adjustment parameters, including the dollar exchange rate and inflation rate, were developed in 2021. We assume a discount rate of 5%. Bangladesh Bank’s dollars exchange rate of Tk.105 to Tk.110 will be used to represent the findings at all estimates [80]. According to the Bangladesh Bank, the inflation rate will also be adjusted with all of our estimates [81]. We will follow the process for four high-risk groups.

### Data safety monitoring plan (DSMP)

The data collected from the participants will be treated with confidentiality, utilizing a unique identification code. The privacy of participants’ data will be ensured, and all data gathered during the research will be maintained in strict confidentiality, with no sharing of information with any other parties. Additionally, strict controls will be implemented to regulate access to the data forms, and strict adherence to preserving data confidentiality will be upheld. The survey responses provided by participants will be treated with strict confidentiality, ensuring that no personal identifying information, including participant names, will be shared.

## Discussion

While research indicates that influenza imposes a significant disease burden and economic costs on high-risk populations in low- and middle-income countries (LMICs), including Bangladesh, influenza vaccination has not received adequate attention in these regions until 2023 [9, 15, 16, 44, 82]. Similar to other low- and middle-income countries (LMICs), Bangladesh has experienced low adoption of influenza vaccines and a dearth of country-specific evidence regarding the effectiveness and cost-effectiveness of vaccination programs targeting high-risk groups [10, 16].

In order to address the existing data gaps and support the development of a national influenza vaccination policy, the present study aims to evaluate the acceptability, cost-effectiveness, and necessary infrastructure for implementing a seasonal influenza vaccination program that targets explicitly high-risk populations in Bangladesh. The study will be carried out in three tertiary-level teaching hospitals situated in Bangladesh. In this study, we will employ the health belief model (HBM) framework to assess the acceptability, health beliefs, barriers, and intention to receive the influenza vaccine. To assess the efficacy of the influenza vaccination, we will offer the vaccine for free to study participants and then follow up with them biweekly for one year. We will also employ a deterministic Markov decision-analytic model to evaluate the cost-effectiveness of facility-based vaccination in Bangladesh.

In early 2019, the WHO published a Global Influenza Strategy for the period of 2019–2030 with the aim of safeguarding individuals across all nations against the potential risks posed by influenza [83]. In order to effectively prevent and control influenza and attain a significant level of progress by the year 2030, it is imperative to prioritize the promotion and implementation of influenza vaccination strategies among high-risk populations, particularly in low- and middle-income countries such as Bangladesh.

One notable limitation of this study is its exclusive focus on two tertiary-level healthcare facilities. In future research, it is strongly recommended to broaden the scope by incorporating a more diverse range of healthcare facilities to enhance the generalizability of the findings.

This study protocol will provide useful data on high-risk groups’ willingness to receive the influenza vaccine, including barriers to vaccination, and will assess whether a facility-based vaccination is cost-effective. The study’s key findings will provide the NITAG and IEDCR, MoH&FW government of Bangladesh, with country-specific data on the cost-effectiveness of facility-based influenza vaccination, supporting advocacy for a national program targeting high-risk groups. Secondly, the data obtained from this study will also help to inform effective interventions targeting high-risk groups for improving influenza vaccine uptake utilizing evidence generated from a vaccine acceptance survey. Finally, the study findings will assist policymakers in designing and implementing a viable influenza vaccination program by estimating the direct and indirect costs of such a programme.

## Data Availability

Not applicable (For the purposes of this article, data sharing does not apply as no datasets have been produced or analyzed).

## References

[1] Nichol KL (2011). Cost-effectiveness and socio-economic aspects of childhood influenza vaccination. Vaccine.

[2] Simonsen L (1999). The global impact of influenza on morbidity and mortality. Vaccine.

[3] Bhuiyan MU, Luby SP, Alamgir NI, Homaira N, Mamun AA, Khan JA, Abedin J, Sturm-Ramirez K, Gurley ES, Zaman RU (2014). Economic burden of influenza‐associated hospitalizations and outpatient visits in B angladesh during 2010. Influenza Other Respir Viruses.

[4] World Health Organization. WHO launches new global influenza strategy. Available at: https://www.who.int/news/item/11-03-2019-who-launches-new-global-influenza-strategy. Accessed 27 January 2022. [ ].

[5] Coleman BL, Fadel SA, Fitzpatrick T, Thomas SM (2018). Risk factors for serious outcomes associated with influenza illness in high-versus low‐and middle‐income countries: systematic literature review and meta‐analysis. Influenza Other Respir Viruses.

[6] Mertz D, Kim TH, Johnstone J, Lam P-P, Kuster SP, Fadel SA, Tran D, Fernandez E, Bhatnagar N, Loeb M. Populations at risk for severe or complicated influenza illness: systematic review and meta-analysis. BMJ 2013, 347. 10.1136/bmj.f5061 PMC3805492 23974637

[7] Organization WH (2012). Vaccines against influenza WHO position paper—November 2012. Wkly Epidemiol Record = Relevé épidémiologique Hebdomadaire.

[8] Hirve S, Lambach P, Paget J, Vandemaele K, Fitzner J, Zhang W (2016). Seasonal influenza vaccine policy, use and effectiveness in the tropics and subtropics–a systematic literature review. Influenza Other Respir Viruses.

[9] Ortiz JR, Perut M, Dumolard L, Wijesinghe PR, Jorgensen P, Ropero AM, Danovaro-Holliday MC, Heffelfinger JD, Tevi-Benissan C, Teleb NA (2016). A global review of national influenza immunization policies: analysis of the 2014 WHO/UNICEF Joint reporting form on immunization. Vaccine.

[10] Hirve S, Organization WH. Seasonal influenza vaccine use in low and middle income countries in the tropics and subtropics: a systematic review. World Health Organization; 2015.

[11] Sibanda M, Meyer JC, Godman B, Burnett RJ (2023). Low influenza vaccine uptake by healthcare workers caring for the elderly in South African old age homes and primary healthcare facilities. BMC Public Health.

[12] Chen H, Li Q, Zhang M, Gu Z, Zhou X, Cao H, Wu F, Liang M, Zheng L, Xian J (2022). Factors associated with influenza vaccination coverage and willingness in the elderly with chronic diseases in Shenzhen, China. Hum Vaccines Immunotherapeutics.

[13] Wiley KE, Massey PD, Cooper SC, Wood NJ, Ho J, Quinn HE, Leask J (2013). Uptake of influenza vaccine by pregnant women: a cross-sectional survey. Med J Aust.

[14] Marrie RA, Walld R, Bolton JM, Sareen J, Patten SB, Singer A, Lix LM, Hitchon CA, Marriott JJ, El-Gabalawy R (2021). Uptake of influenza vaccination among persons with inflammatory bowel disease, multiple sclerosis or rheumatoid arthritis: a population-based matched cohort study. Can Med Association Open Access J.

[15] Ortiz JR, Neuzil KM (2019). Influenza immunization in low-and middle-income countries: preparing for next-generation influenza vaccines. J Infect Dis.

[16] Ott JJ, Klein Breteler J, Tam JS, Hutubessy RC, Jit M, de Boer MR (2013). Influenza vaccines in low and middle income countries: a systematic review of economic evaluations. Hum Vaccines Immunotherapeutics.

[17] Ahmed M, Aleem MA, Roguski K, Abedin J, Islam A, Alam KF, Gurley ES, Rahman M, Azziz-Baumgartner E, Homaira N (2018). Estimates of seasonal influenza‐associated mortality in Bangladesh, 2010‐2012. Influenza Other Respir Viruses.

[18] Azziz-Baumgartner E, Alamgir A, Rahman M, Homaira N, Sohel BM, Sharker M, Zaman RU, Dee J, Gurley ES, Mamun AA (2012). Incidence of influenza-like illness and severe acute respiratory infection during three influenza seasons in Bangladesh, 2008–2010. Bull World Health Organ.

[19] Hassan MZ, Shirin T, Rahman M, Alamgir A, Jahan N, Al Jubayer Biswas MA, Khan SH, Basher MAK, Islam MA, Hussain K (2022). Seasonal influenza vaccine uptake among healthcare workers in tertiary care hospitals, Bangladesh: study protocol for influenza vaccine supply and awareness intervention. BMC Public Health.

[20] Organization WH (2005). Health conditions for travellers to Saudi Arabia pilgrimage to Mecca (Hajj). Wkly Epidemiol Record = Relevé épidémiologique Hebdomadaire.

[21] BARBERIS I, MYLES P, Ault S, Bragazzi N, Martini M (2016). History and evolution of influenza control through vaccination: from the first monovalent vaccine to universal vaccines. J Prev Med Hyg.

[22] Gasparini R, Amicizia D, Lai PL, Bragazzi NL, Panatto D (2014). Compounds with anti-influenza activity: present and future of strategies for the optimal treatment and management of influenza. Part I: Influenza life-cycle and currently available drugs. J Prev Med Hyg.

[23] Peasah SK, Azziz-Baumgartner E, Breese J, Meltzer MI, Widdowson M-A (2013). Influenza cost and cost-effectiveness studies globally–a review. Vaccine.

[24] Dilokthornsakul P, Lan LM, Thakkinstian A, Hutubessy R, Lambach P, Chaiyakunapruk N. Economic evaluation of seasonal influenza vaccination in elderly and health workers: a systematic review and meta-analysis. EClinicalMedicine 2022, 47. 10.1016/j.eclinm.2022.101410 PMC9046113 35497069

[25] Zaman RU, Alamgir A, Rahman M, Azziz-Baumgartner E, Gurley ES, Sharker MAY, Brooks WA, Azim T, Fry AM, Lindstrom S (2009). Influenza in outpatient ILI case-patients in national hospital-based surveillance, Bangladesh, 2007–2008. PLoS ONE.

[26] Bangladesh Bureau of Statistics.Statistical Yearbook of Bangladesh. 2020. Available at: http://www.bbs.gov.bd/site/page/29855dc1-f2b4-4dc0-9073-f692361112da/Statistical-Yearbook. Accessed 27 January 2022. In.

[27] Incepta Vaccine Ltd.Influvax. Available at: http://inceptavaccine.com/product-details.php?pid=influvax. Accessed 16 November 2022.

[28] Sanofi.Vaxigrip. Available at: https://www.mims.com/philippines/drug/info/vaxigrip?type=full. Accessed 16 November 2022.

[29] Directorate General of Health Services (DGHS). Ministry of Health and Family Welfare (MOHFS). Local Health Bulletin;Khulna Medical College Hospital. Available at: http://103.247.238.81/webportal/pages/rtlhb.php?type=5 (Access date, November 11, 2022).

[30] Directorate General of Health Services (DGHS). Ministry of Health and Family Welfare (MOHFS). Local Health Bulletin. Rajshahi Medical College, Rajshahi. Available at: http://103.247.238.81/webportal/pages/rtlhb.php?type=5 (Access date, November 11, 2022).

[31] Maldonado YA, O’Leary ST, Ardura MI, Banerjee R, Bryant KA, Campbell JD, Caserta MT, John CC, Gerber JS, Kourtis AP. Recommendations for prevention and control of influenza in children, 2021–2022. Pediatrics 2021, 148(4). 10.1542/peds.2021-053744 34493537

[32] World Health Organization. Summary of WHO position papers - recommendations for routine immunization.Available at: https://www.who.int/immunization/policy/Immunization_routine_table1.pdf. Accessed 27 January 2022. In.

[33] Jalalabad Ragib Rabeya Medical College &, Hospital S. Access date, November 11,. Available at: http://www.jrrmc.edu.bd/ (2022).

[34] Research NIoP (2020). Training - NIPORT, Health Mo, Family Welfare, ICF: Bangladesh Demographic and Health Survey 2017-18. In. Dhaka.

[35] Sultana M, Mahumud RA, Sarker AR (2017). Burden of chronic illness and associated disabilities in Bangladesh: evidence from the Household Income and Expenditure Survey. Chronic Dis Translational Med.

[36] Champion VL, Skinner CS (2008). The health belief model. Health Behav Health Education: Theory Res Pract.

[37] Cheney MK, John R (2013). Underutilization of influenza vaccine: a test of the health belief model. Sage Open.

[38] Mahmud I, Kabir R, Rahman MA, Alradie-Mohamed A, Vinnakota D, Al-Mohaimeed A (2021). The health belief model predicts intention to receive the covid-19 vaccine in Saudi Arabia: results from a cross-sectional survey. Vaccines.

[39] Mo P, Lau J (2015). Influenza vaccination uptake and associated factors among elderly population in Hong Kong: the application of the Health Belief Model. Health Educ Res.

[40] Nexøe J, Kragstrup J, Søgaard J (1999). Decision on influenza vaccination among the elderly: a questionnaire study based on the Health Belief Model and the Multidimensional Locus of Control Theory. Scand J Prim Health Care.

[41] Hu Y, Wang Y, Liang H, Chen Y (2017). Seasonal influenza vaccine acceptance among pregnant women in Zhejiang Province, China: evidence based on health belief model. Int J Environ Res Public Health.

[42] Ortega-Sanchez IR, Mott JA, Kittikraisak W, Khanthamaly V, McCarron M, Keokhonenang S, Ounaphom P, Pathammavong C, Phounphenghack K, Sayamoungkhoun P (2021). Cost-effectiveness of seasonal influenza vaccination in pregnant women, healthcare workers and adults > = 60 years of age in Lao people’s Democratic Republic. Vaccine.

[43] Edoka I, Kohli-Lynch C, Fraser H, Hofman K, Tempia S, McMorrow M, Ramkrishna W, Lambach P, Hutubessy R, Cohen C (2021). A cost-effectiveness analysis of South Africa’s seasonal influenza vaccination programme. Vaccine.

[44] Costantino C, Vitale F (2016). Influenza vaccination in high-risk groups: a revision of existing guidelines and rationale for an evidence-based preventive strategy. J Prev Med Hyg.

[45] Arsenović S, Trajković G, Pekmezović T, Gazibara T (2022). Validity of the Health Belief Model Applied to Influenza among people with chronic diseases: is it time to develop a new knowledge domain?. PLoS ONE.

[46] Trent MJ, Salmon DA, MacIntyre CR (2021). Using the health belief model to identify barriers to seasonal influenza vaccination among Australian adults in 2019. Influenza Other Respir Viruses.

[47] WHO.WHO surveillance case definitions for ILI and SARI. Available at: https://www.who.int/teams/global-influenza-programme/surveillance-and-monitoring/case-definitions-for-ili-and-sari#:~:text=SARI%20case%20definition,within%20the%20last%2010%20days. Accessed 16 November 2022.

[48] World Health Organization. Information sheet: Observed rate of vaccine reactions, influenza vaccine. Available at:https://www.who.int/vaccine_safety/initiative/tools/Influenza_Vaccine_rates_information_sheet.pdf. Accessed 28 January 2022. [ ].

[49] World Health Organization. Global health estimates: Leading causes of DALYs. Available at:https://www.who.int/data/gho/data/themes/mortality-and-global-health-estimates/global-health-estimates-leading-causes-of-dalys. Accessed 6 February 2022. [ ].

[50] World Health Organization. Global Health Estimates: Life expectancy and leading causes of death and disability. Available at: https://www.who.int/data/gho/data/themes/mortality-and-global-health-estimates. Accessed 6 February 2022. [ ].

[51] WHO methods and data sources for global burden of disease 2000–2019. Global Health Estimates Technical Paper WHO/DDI/DNA/GHE/2020.3.Geneva: World Health Organization; 2020 (https://www.who.int/docs/default-source/gho-documents/global-health-estimates/GlobalBurden_method_2000_2019.pdf).

[52] World Population Prospects: The 2019 revision. New York: United Nations, Department of Economic and Social Affairs, Population Division.; 2019 (https://esa.un.org/unpd/wpp/).

[53] Pecenka C, Parashar U, Tate JE, Khan JA, Groman D, Chacko S, Shamsuzzaman M, Clark A, Atherly D (2017). Impact and cost-effectiveness of rotavirus vaccination in Bangladesh. Vaccine.

[54] Van Minh H, My NTT, Jit M (2017). Cervical cancer treatment costs and cost-effectiveness analysis of human papillomavirus vaccination in Vietnam: a PRIME modeling study. BMC Health Serv Res.

[55] Ministry of Health & Family Welfare. Local Health bulletin.Available at:http://103.247.238.81/webportal/pages/rtlhb_monthly_monitoring.php?type=50. Accessed 27 January 2022.

[56] Biswas MAAJ, Hassan MZ, Monjur MR, Islam MS, Rahman A, Akhtar Z, Chowdhury F, Banu S, Homaira N (2021). Assessment of standard precaution related to infection prevention readiness of healthcare facilities in Bangladesh: findings from a national cross-sectional survey. Antimicrob Stewardship Healthc Epidemiol.

[57] Hassan MZ, Monjur MR, Biswas MAAJ, Chowdhury F, Kafi MAH, Braithwaite J, Jaffe A, Homaira N (2021). Antibiotic use for acute respiratory infections among under-5 children in Bangladesh: a population-based survey. BMJ Global Health.

[58] Samir N, Hassan MZ, Biswas MAAJ, Chowdhury F, Akhtar Z, Lingam R, Banu S, Homaira N (2021). Antibiotic use for febrile illness among under-5 children in Bangladesh: a nationally representative sample survey. Antibiotics.

[59] Demicheli V, Jefferson T, Di Pietrantonj C, Ferroni E, Thorning S, Thomas RE, Rivetti A. Vaccines for preventing influenza in the elderly. Cochrane Database of Systematic Reviews 2018(2). 10.1002/14651858.CD004876.pub4 PMC6491101 29388197

[60] Jefferson T, Rivetti A, Harnden A, Di Pietrantonj C, Demicheli V. Vaccines for preventing influenza in healthy children. *Cochrane Database of Systematic Reviews* 2008(2). 10.1002/14651858.CD004879.pub3 18425905

[61] Rolfes MA, Goswami D, Sharmeen AT, Yeasmin S, Parvin N, Nahar K, Rahman M, Barends M, Ahmed D, Rahman MZ (2017). Efficacy of trivalent influenza vaccine against laboratory-confirmed influenza among young children in a randomized trial in Bangladesh. Vaccine.

[62] Organization WH. Methods for assessing influenza vaccination coverage in target groups. In.: World Health Organization. Regional Office for Europe; 2016.

[63] Group SG-BST (1997). Randomised trial of plasma exchange, intravenous immunoglobulin, and combined treatments in Guillain-Barré syndrome. The Lancet.

[64] World Health O (2020). Flutool plus: WHO seasonal influenza immunization costing tool (SIICT). Pilot version 1.0 edn.

[65] Kadam P, Bhalerao S (2010). Sample size calculation. Int J Ayurveda Res.

[66] Organization WH. Evaluation of influenza vaccine effectiveness: a guide to the design and interpretation of observational studies. 2017.

[67] Lemeshow S, Hosmer DW, Klar J, Lwanga SK, Organization WH (1990). Adequacy of sample size in health studies.

[68] Brooks WA, Zaman K, Lewis KD, Ortiz JR, Goswami D, Feser J, Sharmeen AT, Nahar K, Rahman M, Rahman MZ (2016). Efficacy of a russian-backbone live attenuated influenza vaccine among young children in Bangladesh: a randomised, double-blind, placebo-controlled trial. The Lancet Global Health.

[69] Fell DB, Azziz-Baumgartner E, Baker MG, Batra M, Beauté J, Beutels P, Bhat N, Bhutta ZA, Cohen C, De Mucio B (2017). Influenza epidemiology and immunization during pregnancy: final report of a World Health Organization working group. Vaccine.

[70] Sullivan SG, Price OH, Regan AK (2019). Burden, effectiveness and safety of influenza vaccines in elderly, paediatric and pregnant populations. Therapeutic Adv Vaccines Immunotherapy.

[71] Hair JF, Black WC, Babin BJ, Anderson RE. Multirative data analysis: A global perspective. In.: New Jersey: Pearson Prentice Hall; 2010.

[72] Mangtani P, Cumberland P, Hodgson CR, Roberts JA, Cutts FT, Hall AJ (2004). A cohort study of the effectiveness of influenza vaccine in older people, performed using the United Kingdom general practice research database. J Infect Dis.

[73] Newall A, Jit M, Hutubessy R (2014). Are current cost-effectiveness thresholds for low-and middle-income countries useful? Examples from the world of vaccines. PharmacoEconomics.

[74] Newall AT, Chaiyakunapruk N, Lambach P, Hutubessy RC (2018). WHO guide on the economic evaluation of influenza vaccination. Influenza Other Respir Viruses.

[75] Fesenfeld M, Hutubessy R, Jit M (2013). Cost-effectiveness of human papillomavirus vaccination in low and middle income countries: a systematic review. Vaccine.

[76] Tu H-AT, Woerdenbag HJ, Kane S, Rozenbaum MH, Li SC, Postma MJ (2011). Economic evaluations of rotavirus immunization for developing countries: a review of the literature. Expert Rev Vaccines.

[77] McMorrow ML, Tempia S, Walaza S, Treurnicht FK, Ramkrishna W, Azziz-Baumgartner E, Madhi SA, Cohen C (2019). Prioritization of risk groups for influenza vaccination in resource limited settings–A case study from South Africa. Vaccine.

[78] World Health O (2020). Training for mid-level managers (MLM): module 1: cold chain, vaccines and safe-injection equipment management.

[79] Organization WH (2002). Guidelines for estimating costs of introducing new vaccines into the national immunization system.

[80] Bangladesh Bank. Exchange rate of Taka. Available at: https://www.bb.org.bd/en/index.php/econdata/exchangerate. Accessed 16 November 2022. [ ].

[81] Bangladesh Bank. Current Inflation. Available at: https://www.bb.org.bd/en/index.php/econdata/inflation. Accessed 16 November 2022. [ ].

[82] Restivo V, Costantino C, Bono S, Maniglia M, Marchese V, Ventura G, Casuccio A, Tramuto F, Vitale F (2018). Influenza vaccine effectiveness among high-risk groups: a systematic literature review and meta-analysis of case-control and cohort studies. Hum Vaccines Immunotherapeutics.

[83] Organization WH. Global influenza strategy 2019–2030. 2019.

